# Some Prospective Alternatives for Treating Pain: The Endocannabinoid System and Its Putative Receptors GPR18 and GPR55

**DOI:** 10.3389/fphar.2018.01496

**Published:** 2019-01-08

**Authors:** Raquel Guerrero-Alba, Paulino Barragán-Iglesias, Abimael González-Hernández, Eduardo E. Valdez-Moráles, Vinicio Granados-Soto, Miguel Condés-Lara, Martín G. Rodríguez, Bruno A. Marichal-Cancino

**Affiliations:** ^1^Departamento de Fisiología y Farmacología, Centro de Ciencias Básicas, Universidad Autónoma de Aguascalientes, Aguascalientes, Mexico; ^2^School of Behavioral and Brain Sciences and Center for Advanced Pain Studies, University of Texas at Dallas, Richardson, TX, United States; ^3^Departamento de Neurobiología del Desarrollo y Neurofisiología, Instituto de Neurobiología, Universidad Nacional Autónoma de México, Santiago de Querétaro, Mexico; ^4^Cátedras CONACYT, Departamento de Cirugía, Centro de Ciencias Biomédicas, Universidad Autónoma de Aguascalientes, Aguascalientes, Mexico; ^5^Neurobiology of Pain Laboratory, Departamento de Farmacobiología, Cinvestav, Mexico City, Mexico

**Keywords:** GPR18, GPR55, endocannabinoid system, cannabinoid receptors, pain

## Abstract

**Background:** Marijuana extracts (cannabinoids) have been used for several millennia for pain treatment. Regarding the site of action, cannabinoids are highly promiscuous molecules, but only two cannabinoid receptors (CB_1_ and CB_2_) have been deeply studied and classified. Thus, therapeutic actions, side effects and pharmacological targets for cannabinoids have been explained based on the pharmacology of cannabinoid CB_1_/CB_2_ receptors. However, the accumulation of confusing and sometimes contradictory results suggests the existence of other cannabinoid receptors. Different orphan proteins (e.g., GPR18, GPR55, GPR119, etc.) have been proposed as putative cannabinoid receptors. According to their expression, GPR18 and GPR55 could be involved in sensory transmission and pain integration.

**Methods:** This article reviews select relevant information about the potential role of GPR18 and GPR55 in the pathophysiology of pain.

**Results:** This work summarized novel data supporting that, besides cannabinoid CB_1_ and CB_2_ receptors, GPR18 and GPR55 may be useful for pain treatment.

**Conclusion:** There is evidence to support an antinociceptive role for GPR18 and GPR55.

## Physiology of Pain

### Adaptive Function of Pain

Pain involves unpleasant sensations in response to real or potential tissue damage (Basbaum et al., 2009). Usually, pain unleashes a signal alert to prevent extensive injury by promoting defensive (passive and/or active) actions against the noxious (nociceptive) stimuli. Thus, pain is considered a protective and adaptive mechanism. However, pain may become persistent and pathological without a recognized protective or adaptive mechanism. When this happens, it affects the quality of life of patients and their social environment. Hence, pathological pain is an important medical problem causing distress and disability that requires prompt clinical investigation and treatment (Julius and Basbaum, 2001; Moffat and Rae, 2011). On the other hand, considering that tissue damage is not always the main origin of pain, cognitive perception and somatic sensation should be considered as related but different phenomena. Cognitive perception involves a psychological component frequently related with emotional experiences. Therefore, pain may be cataloged as a subjective event that requires patient awareness (Basbaum and Woolf, 1999; Julius and Basbaum, 2001; Walker and Hohmann, 2005).

### Sensory System: Anatomical and Functional View

The terminal endings of primary afferent neurons whose cell bodies are located in the dorsal root ganglia (DRG) and trigeminal ganglia (TG) are responsible for the transmission of multiple peripheral stimuli (proprioceptive or nociceptive) to the central nervous system (Julius and Basbaum, 2001; Walker and Hohmann, 2005). In the case of nociceptive transmission, two main types of pseudo-unipolar nociceptive neurons are found in those ganglia: (1) non-myelinated small diameter and multimodal C-fibers, which conduct electrical impulses at low speed (∼1 m/s), sensing and transducing thermal, chemical and mechanical stimuli; and (2) thinly myelinated Aδ-fibers that show fast conduction velocity (∼5–30 m/s), sensing mechanical and thermal stimuli (Moffat and Rae, 2011). These primary afferent nociceptive fibers sense the peripheral nociceptive environment and send the nociceptive information to the spinal dorsal horn where they make a synapse with second order neurons, which convey neuronal firing to supraspinal sites where the action potentials are decoded and perceived as pain. At the peripheral level, there are several channels and receptors involved in the initiation of nociceptive transmission, such as the transient receptor potential vanilloid type 1 (TRPV1) channel, tetrodotoxin-resistant (Na^+^-TTXr) voltage-gated sodium (Na^+^) channels, purinergic P_2_X receptors, serotonin (5-HT_3_) channel receptor, and calcium (Ca^2+^) channels, among others.

The nociceptive signal from the peripheral nociceptive fibers is directed toward a second order neuron into the spinal cord, and then the electrical signal is conducted to the brain cortex mainly through the antero-lateral pathway tract where the signal is interpreted as a painful sensation (Snider and McMahon, 1998; Steeds, 2009; Fabbro and Crescentini, 2014). In fact, several sensorial components such as stimuli identification, location, and emotional components are codified in the cortex (Albe-Fessard et al., 1985). The diversity of peripheral and central regions and mechanisms implicated made the control of nociception and pain a complex challenge. Finally, we must keep in mind that nociceptive transmission could be endogenously modulated. For instance, the spinal cord, which is the first relay of nociceptive transmission, could be modulated by diverse neuromodulators (noradrenergic, serotonergic, opioidergic, and oxytocinergic) (for references see Mason, 2001; Vanegas and Schaible, 2004; Loyd and Murphy, 2009; Condés-Lara et al., 2015; Llorca-Torralba et al., 2016) that may diminish or increase the noxious sensation. Nevertheless, these modulatory systems exist along the noxious pathways, including the cortical station. So, the modulation of nociceptive transmission is complex and involves an array of neurotransmitters, neuromodulators and a wide variety of specific and non-specific receptors, which are dysregulated during pathological pain states (Heinricher, 2016).

### Classic Treatments for Pain

**FIGURE 1:**
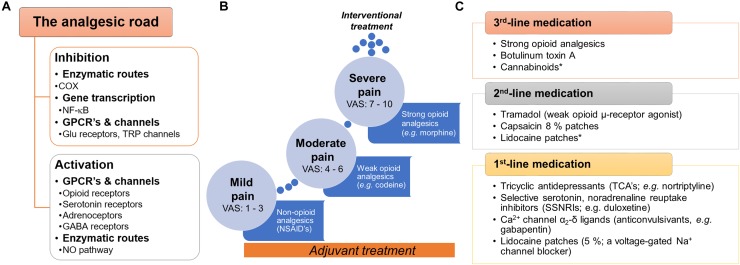
General view of the current pharmacotherapy and guidelines used to treat pain. **(A)** Snapshot of the two main pathways (inhibition or activation) by which analgesic drugs induce pain relief at peripheral, spinal and supraspinal levels. **(B)** To treat pain, the WHO proposed the three-step analgesic ladder. Although primarily for the management of cancer pain, it is also used as a general guideline for the management of acute and chronic non-malignant pain. A key characteristic in this approach is the use of adjuvants^∗∗^ with the primary drug along the pain treatment. **(C)** In the case of neuropathic pain, specific clinical guidelines have been proposed by several international and regional professional associations. Although several recent clinical trials support these guidelines, we need to keep in mind that several factors could limit the applicability in real-world settings (i.e., neuropathic pain is a syndrome caused by diverse etiologies and different clinical manifestations). In general terms, three-line medication has been proposed by several professional associations (including the IASP, EFNS, NICE, and CPS). In all cases, the use of strong opioids is recommended as a 3rd-line medication agent considering the potential risk for abuse, overdose, mortality or misuse. **^∗^**No conclusive efficacy on neuropathic pain treatment. ^∗∗^Adjuvants or co-analgesics are drugs non-specifically designed (or marketed) to treat pain; some examples: glucocorticoids, antidepressants (some SNRIs and TCAs), α_2_-adrenergic agonists (e.g., clonidine) and cannabinoids (including cannabis). COX, cyclooxygenase or prostaglandin-endoperoxide synthase; CPS, Canadian Pain Society; EFNS, European Federation of Neurological Sciences; GPCRs, G protein-coupled receptors, IASP, International Association for the Study of Pain; NICE, National Institute for Health and Care Excellence (of the United Kingdom); WHO, World Health Organization.

Pain treatment can be categorized as pharmacologic and non-pharmacologic. In the first case, there are a variety of druggable targets in both central and peripheral nervous system commonly used for pain treatment. Analgesics are classified as: (i) non-opioid analgesics; (ii) opioid analgesics; and (iii) adjuvant analgesics (Figure 1). The most frequently non-opioid analgesics used are non-steroidal anti-inflammatory drugs (NSAIDs), such as aspirin, ibuprofen and celecoxib. The primary mechanism of action of NSAIDs is through the inhibition of the cyclooxygenase enzymes (COX) by consequently decreasing the action of prostaglandins and their sensitizing properties. Opioid-like drugs, such as morphine, ameliorate pain by modulating the cellular excitability at the supraspinal, spinal and peripheral level through activation of opioid receptors (μ-, δ-, and κ-opioid receptors). Furthermore, opioids could enhance descending inhibitory pathways and modify the sensory and affective components of pain. In the case of adjuvants, local anesthetics (e.g., lidocaine) stop the electrical impulse by blocking voltage-gated sodium (Na^+^) channels. Tricyclic and noradrenaline-reuptake inhibitors act by maintaining and/or augmenting the monoamine levels in descending tracts and anticonvulsants decrease the synaptic transmission affecting neuronal excitability (Basbaum and Woolf, 1999; Sinha et al., 2017).

#### Non-steroidal Anti-inflammatory Drugs

Non-steroidal anti-inflammatory drugs are substances that inhibit a component of the inflammatory cascade and, thence, are an important therapeutic option for non-steroid-based pain treatment. Briefly, these compounds (with exception of acetaminophen) have anti-inflammatory, antipyretic, and analgesic effects by inhibiting COX activity. At this point, we must keep in mind that the COX enzymes have at least three isoforms (COX-1, COX-2 and COX-3) and the non-selective NSAIDs act to block COX-1 and COX-2 indistinctly, favoring gastrointestinal and renal side effects (mediated by COX-1 inhibition). These side effects are particularly common in the elderly, who are most likely to experience chronic pain (Griffin et al., 1991; Buffum and Buffum, 2000; Horl, 2010). To minimize the side effects, selective COX-2 inhibitors have arrived at clinical practice. Unfortunately, several clinical trials have shown that these inhibitors also increase harmful cardiovascular effects (Bhosale et al., 2015).

#### Opioid-Based Treatments

Opioid analgesics act in the central nervous system and are typically prescribed to patients suffering chronic pain refractory to non-opioid treatment. Despite their well-known side effects (sedation, nausea, vomiting, constipation, pruritus and respiratory depression), opioids are widely accepted as effective for acute pain as well as cancer pain. This group of drugs have high abuse liability and are also toxic in elevated doses. For instance, from 1999 to 2014, more than 165,000 persons died of overdose related to opioids in the Unites States. In 2013, an estimated of 1.9 million people abused or were dependent on opioid pain medication (Dowell et al., 2016). Moreover, placebo-controlled trials indicate that, on average, opioids do not result in a clinically significant reduction of chronic pain symptoms (Martell et al., 2007), and even in cases where opioid analgesia is adequate for the individual patient, analgesic effects are typically not maintained during the long-term opioid pharmacotherapy due to pharmacokinetic or pharmacodynamic tolerance (Ballantyne and Shin, 2008; Dumas and Pollack, 2008). Eventually, chronic exposure to opioids results in hyperalgesia (Chu et al., 2008).

#### Antidepressants

Antidepressant drugs have been used as analgesics in chronic pain disorders for decades (Mico et al., 2006). Their pharmacological mechanisms have been associated with the ability to block 5-hydroxytriptamine (serotonin or 5-HT) and noradrenaline re-uptake and consequently with an increase of the activity of the endogenous analgesic system. Tricyclic antidepressants (TCAs) (e.g., amitriptyline and imipramine), tetracyclic antidepressants (TeCAs) (e.g., amoxapine, maprotiline) and the selective serotonin-norepinephrine reuptake inhibitors (SNRIs) (e.g., duloxetine and venlafaxine) are traditionally used to treat chronic pain (Mika et al., 2013). TCAs have been shown to be effective for different neuropathic pain conditions in randomized controlled trials (Finnerup et al., 2010). TCAs are generally reasonably well-tolerated but high doses are associated with a high risk of sudden cardiac death (Ray et al., 2004). The SNRIs duloxetine and venlafaxine have a well-documented efficacy in painful poly-neuropathy (Finnerup et al., 2010). SNRIs are generally well tolerated. However, the most common side-effects reported are nausea, somnolence, dizziness, constipation, anorexia, dry mouth, hyperhidrosis, and sexual dysfunction (Stahl et al., 2005).

#### Anticonvulsants

Gabapentin and pregabalin are anticonvulsants with therapeutic activity against neuropathic pain (Rajapakse et al., 2015). Their analgesic mechanism has been associated to their binding to the α_2_δ_1_ subunit, which in turn blocks voltage-gated calcium (Ca^2+^)-channels at presynaptic sites (Gee et al., 1996) or NMDA receptors at post-synaptic neurons (Chen et al., 2018; Ma et al., 2018). Both drugs are well tolerated but the most common side-effects are somnolence and dizziness, peripheral edema, weight gain, nausea, vertigo, asthenia, dry mouth, and ataxia (Quintero, 2017). Other anticonvulsants used for pain relief are carbamazepine and its analog oxcarbazepine, lamotrigine and valproate. Lamotrigine is effective for central post-stroke pain (Vestergaard et al., 2001) and diabetic neuropathy (Eisenberg et al., 2001), but has failed to relieve pain in patients with multiple sclerosis (Breuer et al., 2007) and neuropathic pain (Silver et al., 2007). Valproate also has a limited role in the treatment of neuropathic pain (Drewes et al., 1994; Otto et al., 2004; Agrawal et al., 2009).

#### Cannabinoids

One alternative for pain treatment came from Asia more than 3000 years ago: marijuana extracts (Li, 1974; Touw, 1981; Jensen et al., 2015). The utility of marijuana-based drugs for treating pain is explained by the existence of an ancient system of cellular control named the endocannabinoid system (ECS). Unfortunately, our knowledge about the physiology of the ECS is only partial (see below). In this review, we summarized novel data supporting that, apart from cannabinoid type-1 (CB_1_) and cannabinoid type-2 (CB_2_) receptors, some putative cannabinoid receptors (i.e., GPR18 and GPR55) may be useful for pain treatment. This should allow researchers to focus their studies on developing endocannabinoid-based options as analgesics and anti-inflammatory drugs.

## Endocannabinoids and Pain

### Endocannabinoid System: Generalities

Despite the ancient and well-known use of cannabis derivatives for pain management, medically recognized use of these compounds has largely subsided due to the lack of knowledge of its molecular pharmacology, its abuse for recreational purposes and additional undesirable effects, such as hypomotility and hypothermia (Crawley et al., 1993), impairments in executive function (Crean et al., 2011) and memory consolidation (Ranganathan and D’Souza, 2006). However, the identification of the major psychoactive component Δ^9^-tetrahydrocannabinol (Δ^9^-THC) (Gaoni and Mechoulam, 1964), and the subsequent isolation of cannabinoid receptors (CB_1_ and CB_2_ receptors, both G-proteins-coupled receptors linked to G*_i/o_* proteins) with high expression levels in the nervous system, led to an explosion of studies exploring the ECS and its regulatory functions in health and disease. Briefly, the ECS consists of endogenous cannabinoids (endocannabinoids, eCBs), cannabinoid receptors, enzymes responsible for synthesis and degradation of eCBs and all genes related to them (Mackie, 2008a,b).

In this context, although several cannabinoids are available, current literature about their potential use for pain treatment remains controversial (Davis, 2014). Indeed, as reviewed by Nurmikko et al. (2007) and Martin-Sanchez et al. (2009), Δ^9^-THC or Δ^9^-THC plus cannabidiol induced relief in only one among six to nine patients (number needed to treat, NNT = 6–9). Moreover, the number needed to harm (NNH) (motor and cognitive dysfunction and altered perception) ranged between five and eight. These data suggest that, apart from its low efficacy, Δ^9^THC could have a narrow therapeutic index. Nevertheless, the above cannabimimetic effects seem to be mainly mediated by CB_1_ receptor activation, suggesting that other parts of the ECS could be druggable to treat pain. In addition, one of the physiological functions attributed to the eCBs is to suppress pain (Walker and Huang, 2002).

#### Endogenous Cannabinoids

**FIGURE 2:**
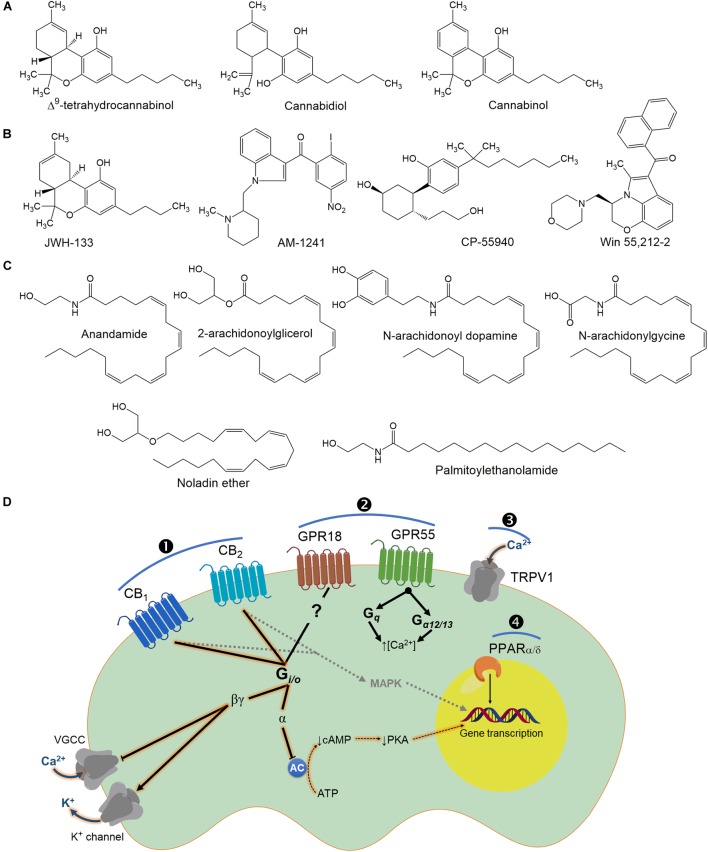
Chemical structures of some plant **(A)**, synthetic cannabinoids **(B)** and endocannabinoids **(C)** that bind to cannabinoid receptors **(D)**. It is interesting to note that cannabinoids could activate intracellular pathways by direct activation of its receptors (\protect❶ and ❷) or modulate other family receptors (\protect❸ and ❹), which contribute to the biological effect of these molecules (particularly for the endocannabinoids). In general terms, classic cannabinoid receptors (CB_1_ and CB_2_) are GPCRs, which are canonically coupled to G*_i/o_* proteins. Consequently, under CB_1/2_ receptors: (i) a decrease of adenylyl cyclase (AC) activity; (ii) an inactivation of Ca^2+^ channels; and (iii) activation of inwardly rectifying K^+^ channels are achieved. These are signal transduction systems associated with inhibition of neurotransmitter release. The inhibition of AC occurs *via* activation of Gα*_i_*-mediated signaling whereas Gα*_o_*-activation results in inhibition of voltage-dependent Ca^2+^ channels (VDCCs) through the release of associated βγ subunits (apparently CB_2_ receptors are ineffective, compared with CB_1_, for shifting ionic currents via βγ subunits). In addition to PKA inhibition, CB_1/2_ receptor signaling also leads to the downstream activation of MAPK which can regulate nuclear transcription factors and consequently expression of several genes. Note that GPR18 seems to be coupled to G*_i/o_* proteins, whereas GPR55 has been associated with an increase of intracellular Ca^2+^ via G_α12/13_. In the case of TRPV1 channels (a non-selective cation channel for Ca^2+^, Mg^2+^, and Na^+^ ions), it is well-known that agonist can be used rationally for the treatment of pain considering that this channel under constant activation desensitizes the nociceptive neuron. Finally, although not fully investigated, cannabinoid compounds could also activate PPAR_α/δ_, which are involved in pain modulation and transmission.

The first eCB isolated in the brain was *N*-arachidonoyl ethanolamide (AEA), or anandamide (a name taken from the Sanskrit word Ananda, which means “bliss, joy,” and amide) (Devane et al., 1992; Figure 2). AEA is a fatty acid neuromodulator derived from the non-oxidative metabolism of arachidonic acid (AA). The second endocannabinoid identified was 2-arachidonoyl glycerol (2-AG) (Mechoulam et al., 1995; Sugiura et al., 1995). As the search for endogenous Δ^9^-THC-like compounds continued, other bioactive lipids were extracted from animal tissues. These include noladin ether (Hanus et al., 2001), virodhamine (Porter et al., 2002) and *N*-arachidonoyl dopamine (NADA) (Huang et al., 2001).

The most widely investigated eCBs are anandamide and 2-AG. Indeed, anandamide is present in about 170-fold lower levels of brain tissue than 2-AG (Stella et al., 1997), and both lipidic derivatives activate cannabinoid CB_1_ and CB_2_ receptors. Certainly, anandamide shows preferential affinity for CB_1_ (K_i_ = 89 nM) compared to CB_2_ (K_i_ = 371 nM) receptors (Gauldie et al., 2001), whereas 2-AG is considered a full agonist at both CB_1_ and CB_2_ receptors (Sugiura and Waku, 2000). Nevertheless, it has been shown that AEA could activate the vanilloid type-1 receptor (TRPV1), which contributes to the many non-CB_1_-mediated effects (Zygmunt et al., 1999; Smart et al., 2000). Furthermore, AEA and other eCBs (palmitoylethanolamide [PEA] and oleylethanolamide [OEA]) also are agonists of the peroxisome proliferator-activated receptor α (PPARα) (Fu et al., 2003; Bouaboula et al., 2005; Lo Verme et al., 2005). PEA also has a well-established role in pain modulation and inflammation in rodents (Jaggar et al., 1998; Calignano et al., 2001; Lo Verme et al., 2005; D’Agostino et al., 2007; González-Hernández et al., 2015), whereas in humans PEA treatment seems to relieve neuropathic pain (Calabro et al., 2010; Conigliaro et al., 2011; Gatti et al., 2012).

The eCBs are atypical neurotransmitters and/or neuromodulators. They are not stored in synaptic vesicles and are not released from presynaptic terminals via an exocytotic mechanism. In fact, their precursors exist in the cell membrane, are cleaved by specific enzymes “on demand” depending on intracellular calcium increase and are released from cells immediately after their production. The synthesis, release and deactivation of the endogenous cannabinoids are tightly regulated processes. As discussion of these processes is beyond the scope of this review, the interested reader is referred to several reviews on the topic (Howlett, 2002; Piomelli, 2003; Simon and Cravatt, 2006; Okamoto et al., 2007; Ueda et al., 2011; Luchicchi and Pistis, 2012).

#### Cannabinoid Receptors

To date, there are two known cannabinoid receptors that are part of the ECS, the CB_1_ and CB_2_ receptors. These receptors belong to the 7-transmembrane G-protein coupled receptors (GPCRs) primarily coupled to G*_i/o_* proteins that inhibit adenylyl cyclase (AC) and increase mitogen-activated protein kinase (MAPK) activity downstream of β-arrestin (Howlett, 2002; Vasileiou et al., 2013). Activation of these receptors triggers the inwardly rectifying potassium (K^+^)-channels and A-type potassium (K^+^)-channel currents and inhibits N-Type and P/Q type calcium (Ca^2+^)-channel activity (Demuth and Molleman, 2006). The CB_2_ receptor is also negatively coupled to adenylyl cyclase but it seems not to be coupled to calcium (Ca^2+^)-channels (Felder et al., 1995). However, CB_1_ receptors can also interact with G*_s_* and G*_q/11_* under certain conditions and with certain agonists (Mackie, 2005, 2008b). In addition, a pair of orphan-related receptors (GPR18 and GPR55) is also described as cannabinoid putative receptors.

##### CB_1_ receptor expression

The CB_1_ receptor is highly expressed in the cortex, cerebellum and associational cortical regions of neocortex (Glass et al., 1997). It is also expressed in the spinal dorsal horn (Sanudo-Pena et al., 1999) and in DRG neurons (Hohmann and Herkenham, 1999; Salio et al., 2002; Walker and Hohmann, 2005). Autonomic nerve terminals express CB_1_ receptors (Ishac et al., 1996; Vizi et al., 2001), which negatively modulate the sympathetic tone (Marichal-Cancino et al., 2013). Low levels of these receptors have been reported in the adrenal gland, thymus, heart, bone marrow, tonsils, prostrate, uterus, ovary and lung (Galiegue et al., 1995; Rice et al., 1997). A key characteristic of this receptor is the formation of heterodimers, suggesting that intracellular signaling could change under different conditions (Callen et al., 2012; Laprairie et al., 2012; Straiker et al., 2012).

##### CB_2_ receptor expression

The CB_2_ receptor is mostly expressed on cells of the immune system and spleen (Munro et al., 1993; Galiegue et al., 1995; Di Marzo et al., 2004). A few studies have found CB_2_ immunoreactivity expression in glial and neuronal cells in some areas of the rodent brain (Gong et al., 2006; Onaivi et al., 2006), but this expression remains controversial (Hohmann and Herkenham, 1999; Salio et al., 2002; Walker and Hohmann, 2005). Notably, nerve injury and inflammation upregulate expression of CB_2_ receptors in neurons and microglia (Beltramo et al., 2006; Rahn and Hohmann, 2009; Sagar et al., 2009; Hsieh et al., 2011). Furthermore, some studies have demonstrated the presence of CB_2_ receptors in the DRG and afferent fibers in the spinal dorsal horn (Ross et al., 2001; Anand et al., 2008).

### Role of CB_1_ and CB_2_ Receptors on Primary Afferent Neurons

DRG neurons express CB_1_ receptors (Hohmann and Herkenham, 1999; Ross et al., 2001; Price et al., 2003). This receptor is synthesized in the cell neuronal bodies and inserted on both central and peripheral terminals (Hohmann and Herkenham, 1999; Hohmann et al., 1999). CB_1_ receptors are mainly expressed in myelinated fibers of DRG neurons (Hohmann and Herkenham, 1999; Salio et al., 2002; Bridges et al., 2003) and also co-localize with CGRP, TRPV1 and IB4 (Hohmann and Herkenham, 1999; Hohmann et al., 1999; Ahluwalia et al., 2000; Bridges et al., 2003; Veress et al., 2013).

Nerve injury enhances CB_1_ receptor expression in the DRG and spinal cord (Lim et al., 2003; Wang et al., 2007; Shiue et al., 2017) and other brain areas related with the emotional component of pain (Knerlich-Lukoschus et al., 2011). These data give an anatomical basis for the involvement of CB_1_ receptors in modulating neuropathic pain. In this regard, it has been shown that systemic and local administration of CB_1_ receptor agonists produce anti-nociceptive effects in neuropathic pain models (Herzberg et al., 1997; Fox et al., 2001; Bridges et al., 2003; Yu et al., 2010). Moreover, deletion of CB_1_ receptors in peripheral (but not at spinal or supraspinal level) nociceptors reduced analgesia by local or systemic (but no intrathecal) CB_1_ receptor agonists (Agarwal et al., 2007). Thus, CB_1_ receptors located at primary afferent neurons constitute the prime target for producing cannabinoid analgesia.

Some of the peripheral antinociceptive effects of cannabinoids may occur through interaction with another receptor system. In this regard, an early work in rat nodose ganglion neurons showed that cannabinoid agonists inhibited 5-HT-induced currents in a concentration-dependent manner. The inward current was sensitive to the serotonin (5-HT_3_) receptor antagonist MDL72222, suggesting a cannabinoid-mediated inhibition of serotonin (5-HT_3_) currents (Fan, 1995). Later, *in vivo* experiments demonstrated that application of CB_1_ and CB_2_ receptor agonists attenuated the activity of rat peripheral (5-HT_3_) receptors on the terminals of cardiopulmonary afferent C-fibers (Godlewski et al., 2003) through an allosteric interaction at a (5-HT_3_) modulatory site (Barann et al., 2002). Moreover, the inhibitory effects of cannabinoids may occur through a synergistic action with opioid receptors and their signal transduction pathways (Pugh et al., 1996; Smith et al., 1998; Manzanares et al., 1999; Massi et al., 2003; Scavone et al., 2013) or by a cannabinoid-mediated increase in opioid peptide synthesis and release of endogenous opioids such as enkephalins and dynorphins (Corchero et al., 1997a,b; Valverde et al., 2001).

The use of cannabinoid agonists as analgesic drugs is limited due to adverse effects in the CNS (Clermont-Gnamien et al., 2002; Attal et al., 2004; Turcotte et al., 2010). However, since it has been demonstrated that CB_1_ receptors are expressed at primary afferent neurons (Agarwal et al., 2007), the synthesis of CB_1_ receptor agonists with limited CNS penetration is under development (Clapper et al., 2010; Yu et al., 2010).

The molecular mechanisms by which the CB_1_ receptor has peripheral antinociceptive effects are not completely understood. It is known that CB_1_ receptor, coupled to G*_i/o_* protein, can modulate several cellular mechanisms, all of which can reduce the excitability of neurons (e.g., opening of inward rectifying potassium (K^+^)-channels and A-type potassium (K^+^)-channels, and inhibiting N-Type and P/Q type calcium (Ca^2+^)-channels) (Demuth and Molleman, 2006). Moreover, there are several studies showing that cannabinoids can modulate the activity of transient receptor potential (TRP) channels, which are implicated in the modulation of pain processing. For example, multiple studies have shown that activation of the CB_1_ receptor suppresses capsaicin-induced hyperalgesia in afferent neurons (Ko and Woods, 1999; Li et al., 1999; Johanek et al., 2001; Millns et al., 2001; Johanek and Simone, 2004; Santha et al., 2010). However, there are controversial findings regarding the effects of CB_1_ receptor agonists on TRPV1 channels, because the CB_1_ receptor agonist anandamide exerts dual effects on afferent neurons, depending on the concentration used (Ross, 2003; Evans et al., 2004; Sousa-Valente et al., 2014). Specifically, anandamide produces a CB_1_-mediated inhibitory effect at nM concentration, while it exerts a TRPV1-mediated stimulatory effect at higher concentrations (μM) in primary afferent neurons (Tognetto et al., 2001; Roberts et al., 2002; Ross, 2003; Fischbach et al., 2007). A recent study using mouse afferent neurons has shown that activation of CB_1_ receptors inhibit nerve growth factor (NGF)-induced sensitization of TRPV1 (Wang et al., 2014), possibly through multiple signaling pathways, including ERK1/2 and PI3K (Zhuang et al., 2004; Stein et al., 2006; Zhu and Oxford, 2007).

The analgesic action of cannabinoids may be mediated by the presynaptic inhibition of neurotransmitter release in sensory neurons. For example, presynaptic CB_1_ receptors inhibit CGRP and substance P (SP) release from trigeminal sensory nerves (Akerman et al., 2004; Oshita et al., 2005). Moreover, CB_1_ receptor agonists reduce voltage-activated Ca^2+^ current in DRG neurons (Ross et al., 2001). On the other hand, it is possible that even more important than peripheral actions, cannabinoids induce analgesia by interfering with circuitry in the rostral ventromedial medulla (RVM) (Meng et al., 1998).

CB_2_ receptors have also been found in nociceptive sensory neurons of rodents (Ross et al., 2001; Merriam et al., 2008; Schuelert et al., 2010) and humans (Anand et al., 2008). Like with CB_1_ receptors, nerve damage upregulates CB_2_ receptors in the superficial laminae of the dorsal horn of the spinal cord and isolated DRG of mice (Wotherspoon et al., 2005) and human beings (Anand et al., 2008).

Although the specific role of the CB_2_ receptor in sensory neurons remains unclear, several functional studies in sensory neurons point to an antinociceptive role (Burston and Woodhams, 2014). For instance, the putative CB_2_ receptor agonist JWH-133 inhibits capsaicin-induced depolarization of the vagus sensory nerve in guinea pigs and humans (Patel et al., 2003). Moreover, JWH-133 reduces the response of wide dynamic range dorsal horn neurons to both innocuous and noxious intensities of mechanical stimuli (Elmes et al., 2004). This compound also attenuates the capsaicin-evoked Ca^2+^ response in DRG neurons in neuropathic rats (Sagar et al., 2009), while GW818646X (other CB_2_ receptor agonist) diminishes capsaicin-induced inward cation currents and elevation of cytoplasmic Ca^2+^ (Anand et al., 2008). Another CB_2_ receptor agonist, A-836339, inhibits von Frey-evoked activity of WDR neurons in neuropathic rats (McGaraughty et al., 2009). Local peripheral injection of the selective CB_2_ receptor agonist AM1241 into the hind paw produces antinociception to thermal stimulation (Malan et al., 2001). AM1241 also inhibits bradykinin-induced mesenteric afferent nerve activity (Hillsley et al., 2007). This effect was absent in CB_2_ knock-out mice and blocked by AM630, a CB_2_ receptor inverse agonist. Local injection of the PEA analog *N*-(4-methoxy-2-nitrophenyl)hexadecanamide induces CB_1_- and CB_2_-dependent antinociception in rats (Roa-Coria et al., 2012). Similar results were observed with GW833972A, another putative CB_2_ receptor agonist (Belvisi et al., 2008). Interestingly, repeated systemic administration of the CB_2_ receptor selective agonist AM1710 suppresses paclitaxel-induced allodynia (Deng et al., 2015). Taken together, the data strongly suggest that CB_1_ and CB_2_ receptors have an antinociceptive role. Despite this evidence, there are few cannabinoid-based drugs currently available for clinical use (see below).

### CB_1_ and CB_2_-Based Treatment for Pain

A randomized, placebo-controlled, double-blinded crossover design was used to examine the effect of cannabinoids on pain. Low, medium, and high doses of smoked cannabis (respectively 2, 4, and 8% Δ^9^-THC by weight) did not modify capsaicin-induced pain assessed in 15 healthy volunteers 5 min after exposure (Wallace et al., 2007). In contrast, the medium dose of Δ^9^-THC diminished capsaicin-induced pain 45 min after cannabis exposure. Of note, these authors found that a high dose of cannabis increased capsaicin-induced pain (Wallace et al., 2007). Similar results have been reported with a high dose of nabilone (an oral synthetic cannabinoid Δ^9^-THC analog) on 41 patients with postoperative pain (Beaulieu, 2006). Another study evaluated cannabis extract capsules (20 mg of Δ^9^-THC) in 18 healthy female volunteers (Kraft et al., 2008). Treatment with Δ^9^-THC was not able to reduce pain induced by capsaicin, electrical stimulation or sunburn. Taken together, it seems that Δ^9^-THC is not effective for acute pain. A similar conclusion was reached after analyzing a total of 611 patients in seven well-designed studies (Stevens and Higgins, 2017).

Although the effects of cannabinoids in the acute pain setting seem to be disappointing, results of clinical trials evaluating cannabinoids in chronic pain are much more promising (see Table 1). The conditions causing chronic pain varied between studies and included neuropathy (chemotherapy, diabetes, human immunodeficiency virus [HIV]), cancer, fibromyalgia, multiple sclerosis and rheumatoid arthritis (Whiting et al., 2015). Sativex (containing Δ^9^-THC:cannabidiol [CBD] in an approximate 1:1 ratio [oral spray]) reduced neuropathic pain in patients with unilateral neuropathic pain (Berman et al., 2004; Nurmikko et al., 2007; Langford et al., 2013; Serpell et al., 2014). Likewise, treatment with smoked cannabis diminished pain in patients with multiple sclerosis (Rog et al., 2005; Corey-Bloom et al., 2012), neuropathic pain (Wilsey et al., 2013) and diabetic neuropathy (Wallace et al., 2015). In contrast, sativex was ineffective in relieving chemotherapy-induced neuropathic pain (Lynch et al., 2014). Oral administration of dronabinol, a synthetic Δ^9^-THC analog, modestly reduced central pain in patients with multiple sclerosis (Svendsen et al., 2004). Nabilone, another synthetic Δ^9^-THC analog, diminished neuropathic pain in diabetic patients (Toth et al., 2012). Oral administration of Δ^9^-THC (ECP002A) reduced pain in patients with progressive multiple sclerosis. Drug dosage was well tolerated and had a stable pharmacokinetic profile (van Amerongen et al., 2017). Nabilone is also effective in patients with medication overuse headache (Pini et al., 2012). In contrast, nabilone did not reduce pain in patients with fibromyalgia (Skrabek et al., 2008).

**Table 1:** Studies about the antinociceptive effects of CB1 and CB2 receptor agonists in different pain models.

Pain model	Drug treatment and dose	Behavioral readout	Route	Results	Proposed mechanisms of action	Reference
Partial SNL	WIN 55,212-2 0.3–10 mg/kg CP-55,940 0.03–1 mg/kg HU-210 0.001–0.03 mg/kg	Mechanical hyperalgesia Thermal hyperalgesia Tactile allodynia	s.c. or i.t.	They produce complete reversal of mechanical hyperalgesia with catalepsy Only WIN 55,212-2 reversed tactile allodynia and thermal hyperalgesia in this model	Via activation of CB1 receptors in both CNS and in the periphery	Herzberg et al., 1997; Fox et al., 2001; Bridges et al., 2003
SNL or carrageenan model	AZ11713908 0.6–1.2 μmol/kg	Thermal and mechanical hyperalgesia	s.c.	Robust analgesia in both models	Likely via peripheral activation of CB1 receptor	Yu et al., 2010
Mechanical stimulation, formalin or capsaicin models, in mice that lacked CB1 receptor specifically in primary nociceptors	Endocannabinoids (AEA and arachidonic acid)	Thermal and mechanical hyperalgesia		The nociceptor-specific loss of CB1 receptor substantially reduced the analgesia produced by local and systemic but no intrathecal, delivery of cannabinoids	Via CB1 receptors expressed on the peripheral terminals of nociceptors	Agarwal et al., 2007
SNL, carrageenan, LPS or CIA model	URB937 1 mg/kg URB597 10 mg/kg PF-3845 0.1-10 μg/kg	Thermal and mechanical hyperalgesia, tactile allodynia	i.p. or i.t.	Attenuation of hyperalgesia and partial reduction of allodynia	Suppresses FAAH activity and increases AEA levels	Clapper et al., 2010; Kinsey et al., 2011; Booker et al., 2012
FCA, partial SNL, tail flick, hot plate or incision model of postoperative pain	GW405833 0.3–30 mg/kg	Mechanical hyperalgesia and tactile allodynia	i.p.	Elicits potent and efficacious antihyperalgesic effects in rodent models of neuropathic, incisional and chronic inflammatory pain	Via activation of CB2 receptors	Valenzano et al., 2005
FCA, chronic constriction injury, incision model of postoperative pain or knee joint osteoarthritic pain	A796260 11–35 mg/kg	Thermal and tactile allodynia	i.p.	Analgesic activity in all pain models	Via activation of CB2 receptors	Yao et al., 2008
Partial SNL or carrageenan model	JWH133 50–100 nmol/mouse	Tactile allodynia	i.t., i.p. or local	Reverses partial sciatic nerve ligation-induced mechanical allodynia in mice.	Via activation of central CB2 receptors	Patel et al., 2003; Elmes et al., 2004; Yamamoto et al., 2008; Sagar et al., 2009
SNL, Formalin, Carrageenan, FCA or intradermal capsaicin	AM1241 0.03–6 mg/kg	Tactile and thermal allodynia, mechanical hyperalgesia and nocifensive response	i.v., i.p. or i.pl.	Analgesic effects in all pain models	Via activation of peripheral CB2 receptors	Malan et al., 2001, 2002; Ibrahim et al., 2003; Quartilho et al., 2003; LaBuda et al., 2005; Beltramo et al., 2006; Hillsley et al., 2007; Yao et al., 2008
Formalin model or postoperative pain	HU308 30, 50 mg/kg	Nocifensive response and actile allodynia	i.p.	Reduces blood pressure, blocks defecation, and elicits anti-inflammatory and peripheral analgesic activity	Via activation of CB2 receptors	Hanus et al., 1999; LaBuda et al., 2005
FCA or chronic constriction injury	GW842166X 0.1–0.3, 15 mg/kg	Mechanical hyperalgesia	p.o.	Very potent analgesic in inflammatory and neuropathic pain models	Potent and highly selective full agonist at the CB2 receptor	Clayton et al., 2004; Giblin et al., 2007; Anand et al., 2008
SNL	A836339 1–3 μmol/kg	Tactile allodynia	i.v.	Reduces both spontaneous and von Frey-evoked firing of WDR neurons in neuropathic rats	Via activation of spinal and peripheral CB2 receptors	McGaraughty et al., 2009
Paclitaxel-neuropathic pain	AM1710 0.1–10 mg/kg	Mechanical and thermal allodynia	i.p.	Suppresses allodynia generated by paclitaxel without central side effects	Via activation of CB2 receptors	Rahn et al., 2011; Deng et al., 2015


*AEA, anandamide; SNP, spinal nerve ligation; FCA, Freud’s complete adjuvant; CIA, collagen-induced arthritis; LPS, lipopolysaccharide; s.c., subcutaneous; i.p, intraperitoneal; i.t., intrathecal; i.v., intravenous; p.o., oral administration; i.pl, intraplantar.*

A limitation to clinical use of cannabinoids for pain is their unfavorable side-effect profile, such as drowsiness, dizziness, speech impediments, memory impairment and confusion. Results of clinical trials with these agents indicate that high dosages are required to attain therapeutic effects and it is difficult to reach these dosages in clinical practice (Turcotte et al., 2010). At doses that prevent subjective effects, some cannabinoids seem to be ineffective for controlling acute pain (Kalliomäki et al., 2013). Several peripherally restricted CB_1_ and CB_2_ receptor agonists have been developed to avoid these side effects (Pertwee, 2009; Yu et al., 2010; Rahn et al., 2011; Yrjola et al., 2013). However, additional research is needed to improve study methodologies including the use of standard formulations and/or dosages, the increase in the number of subjects involved, and the general determination of the safe and effective use of cannabis for the treatment of human pain.

Another interesting area of research has recently focused on the evaluation of the possible synergy between cannabinoids and opioids in the management of pain. A combination of Δ^9^-THC and morphine diminished experimental pain in healthy volunteers (Roberts et al., 2006). Furthermore, dronabinol combined with opioids relieved chronic pain in patients (Narang et al., 2008).

In the last years, pain research has focused on the inhibition of the enzymes playing a role in EC metabolism and the elevation of the EC tonus locally. Special emphasis is given on multi-target analgesia compounds, where one of the targets is the EC degrading enzyme. Dual FAAH^1^ /TRPV1 blockers, such as *N*-arachidonoyl-serotonin (AA-5-HT) and OMDM198, are effective in animal studies, but this multi-target strategy has not yet reached the clinic (Maione et al., 2007, 2013; Morera et al., 2009; Costa et al., 2010; Malek et al., 2015).

Importantly, cannabinoids interact (apart from CB_1_ and CB_2_) with several other pharmacological receptors, including the cannabinoid putative receptors GPR18 and GPR55 (which have been even suggested as CB_x_ and CB_3_ receptors). It is likely that the contradictory effects observed in clinical trials using *Cannabis sp*.-based treatments (e.g., Δ^9^-THC) may be due to the high promiscuity of cannabinoids for their receptors. Before achieving a clinical benefit from an EC system-based therapy in pain (and other alterations), it is mandatory to detect and understand the physiological and/or pathophysiological role of the cellular targets involved. In this context, we provide an analysis of the potential participation of the putative cannabinoid receptors GPR18 and GPR55 in pain (see below).

## Gpr18 and Gpr55: Potential Targets for Pain Treatment

### GPR55 and GPR18: Generalities

Cannabinoids interact with multiple orphan receptors (Alexander, 2012). Different groups have discussed if G protein-coupled receptor 18 (GPR18) and 55 (GPR55) should be considered as novel cannabinoid receptors (Alexander, 2012; Alexander et al., 2017). Nevertheless, the nomenclature suggested by the Nomenclature Committee of the Union of Basic and Clinical Pharmacology (NC-IUPHAR) Subcommittee on Cannabinoid Receptors (Pertwee et al., 2010) decided that all criteria to consider these as novel cannabinoid receptors remain incomplete and, accordingly, they were classified again as orphan receptors (Alexander et al., 2017). Independently of the official decision, these receptors clearly interact with cannabinoids directly or indirectly. Expression of GPR18 seems to be rich in the testis, spleen, peripheral blood leucocytes and lymph nodes (Gantz et al., 1997; Vassilatis et al., 2003; Rosenkilde et al., 2006). Its expression suggests a potential role in the control of immune system activity (e.g., leucocytes migration) (Burstein et al., 2011) and accordingly inflammation. Moreover, activation of GPR18 by *N*-arachidonoylglycine leads to apoptosis of inflammatory leukocytes (Burstein et al., 2011; Takenouchi et al., 2012), which in turn reduces local inflammation. There is also evidence that activation of GPR18 lowers intraocular pressure in mice (Miller et al., 2016). All these findings suggest a physiological function of NAGly *via* GPR18 in different inflammatory processes.

Knowledge about GPR55 physiology in the nervous system has increased recently (Marichal-Cancino et al., 2017). This receptor has been suggested as a potential therapeutic target in Parkinson’s disease due to a possible alteration on its expression in the basal nuclei (Celorrio et al., 2017), where it is related to procedural memories (Marichal-Cancino et al., 2016). GPR55 is also expressed in the hippocampus, where it has a role in spatial navigation (Marichal-Cancino et al., 2018). Furthermore, it is possible that some antiepileptic actions observed with phytocannabinoids involve the blocking of GPR55 (Kaplan et al., 2017). However, the above is a topic under study and findings are preliminary. Despite all advances in the physiology of GPR55, several actions in different areas of the CNS remain obscure (Marichal-Cancino et al., 2017). Interestingly, PEA (a cannabinoid related compound) is currently used to treat pain and inflammation. Like other cannabinoid related molecules, PEA has a very complex mechanism of action, which includes direct and/or indirect interaction with CB_1_, TRPV1, PPAR, GPR55 and GPR18, among other receptors (Keppel Hesselink et al., 2014). Certainly, PEA has high affinity for GPR55 as a full agonist (Ryberg et al., 2007). Thus, it is necessary to investigate whether GPR55 is involved in the analgesic and anti-inflammatory actions of PEA.

### Actions of GPR18 and GPR55 and Their Potential Role in the Pharmacology of Pain

GPR18 and GPR55 are differentially expressed in the central and peripheral nociceptive systems of rodents and humans, suggesting a potential role in the modulation of nociceptive pathways (DRG TXome Database)^2^(Ray et al., 2018). In general, GPR18 is less studied compared to GPR55 (see below). This is partly due to the fact that signaling mechanisms and endogenous ligands are still controversial (Alexander et al., 2017). GPR18 has been suggested to modulate, depending on the ligand, both G*_αi/o_* and G*_αq/11_* transduction pathways (Console-Bram et al., 2014). In this sense, NAGly is proposed as the endogenous GPR18 ligand (Kohno et al., 2006; McHugh et al., 2010). However, a recent study suggests that NAGly increases Ca^2+^ mobilization and MAPK activity in HAGPR55/CHO cells (Console-Bram et al., 2017). This response is attenuated by ML193 (GPR55 receptor antagonist) suggesting that NAGly-mediated effects depend on GPR55 activation. Moreover, an independent study reported that NAGly does not activate GPR18 receptors (Lu et al., 2013). In support of this, there is a previous observation showing that NAGly does not activate GPR18 (Yin et al., 2009). These discrepancies could be partially explained by the fact that NAGly is also a reversible and non-competitive inhibitor of the glycine transporter type 2 (GlyT2) (Wiles et al., 2006). In line with this, it has been shown that NAGly enhances inhibitory glycinergic transmission synaptic within the superficial dorsal horn by blocking glycine uptake *via* GlyT2 and decreasing excitatory NMDA-mediated synaptic transmission (Jeong et al., 2010).

It has been proposed that both GPR18 and GPR55 could play a role in the modulation of acute and chronic pain (Table 2). In animal models of inflammatory pain, intraplantar NAGly administration attenuates formalin-induced pain (Huang et al., 2001). Moreover, intrathecal administration of NAGly reduces complete Freund’s adjuvant (CFA)-induced mechanical allodynia and thermal hyperalgesia by a CB_1_-independent mechanism (Succar et al., 2007). Additionally, NAGly increases the production of 15-deoxy-Δ^13,14^-prostaglandin J2 and lipoxin A4, leading to a reduction in the migration of inflammatory cells into the area of acute inflammation (Burstein et al., 2011). GPR18 is expressed on human leukocytes, including polymorphonuclear neutrophils (PMN), monocytes, and macrophages and, furthermore, its activation regulates leukocyte trafficking during acute inflammation (Chiang et al., 2015). GPR18 and TRPV1 are expressed in chondrocytes within the deep zone of cartilage in patients with osteoarthritis (OA) (Dunn et al., 2016), suggesting that GPR18 presence in degenerate tissues could be a target for treatment with cannabinoids.

**Table 2:** Possible role of GPR18 and GPR55 receptors in different animal models of pain.

Pain model/specie	Drug treatment	Dose	Route	Outcome	Proposed mechanisms of action	Reference
Formalin/rat	NAGly	275 nmol	i.pl.	Suppression of phase II response	Non-CB1 mediated mechanism	Huang et al., 2001
	CID16020046	10 μM	Intra-ACC	Attenuation of phase II response Reduction of p-ERK in the ACC Attenuation of spinal *c-fos* expression in the spinal cord	Endogenous activation of GPR55 signaling. Modulatory effects of GPR55 signaling in the ACC on the descending pain pathway	Okine et al., 2016
Formalin/mouse	N/T	N/T	N/T	No differences between WT and GPR55^-/-^ mice in mechanical, cold and heat hypersensitivity	Non-GPR55 mediated mechanism	Carey et al., 2017
CFA/rat	NAGly	70–700 nmol	i.t.	Attenuation of mechanical and thermal hyperalgesia	Non-cannabinoid mediated mechanism	Succar et al., 2007
CFA/mouse	N/T	N/T	N/T	Absence of mechanical hyperalgesia in GPR55^-/-^ mice	GPR55 signaling	Staton et al., 2008
Capsaicin/mouse	N/T	N/T	N/T	GPR55^-/-^ and WT mice display comparative levels of capsaicin-evoked nocifensive behavior, mechanical and thermal hyperalgesia	Non-GPR55 mediated mechanism	Carey et al., 2017
PNL/rat PNL/Mouse PNL/Mouse	NAGly N/T N/T	70–700 nmol N/T N/T	i.t. N/T N/T	Reduction of mechanical allodynia Absence of mechanical hyperalgesia in GPR55^-/-^ mice GPR55^-/-^ and WT mice develop similar levels of hypersensitivity to mechanical, heat, and cold stimulation	CB1 and CB2 independent mechanism GPR55 signaling Non-GPR55 mediated mechanism	Vuong et al., 2008 Staton et al., 2008 Carey et al., 2017
CCI/rat	O-1602 AA-5-HT	1–10 mg/kg 100–1000 nM	i.p. i.t.	Pronociceptive properties in neuropathic pain induced by O-1602 (atypical cannabinoid) Upregulation of CB2, GPR18, and GPR55 mRNA in the spinal cord and/or DRG after CCI. Increased pain threshold to mechanical and thermal stimuli following AA-5HT	Pronociceptive role of GPR55. Possible role of GPR18 Involvement of CB2, GPR18 and GPR55 receptors	Breen et al., 2012 Malek et al., 2016
Paclitaxel/mouse	N/T	N/T	N/T	GPR55^-/-^ and WT mice develop similar levels of paclitaxel-induced mechanical and cold allodynia	Non-GPR55 mediated mechanism	Carey et al., 2017
LPI-induced pain/mouse	LPI	2 pmol–6 nmol	i.pl.	WT mice: Sensitization against non-painful and painful mechanical stimuli. GPR55^-/-^ mice: reduction of LPI-induced acute allodynia, attenuation of LPI-induced long-term mechanical hyperalgesia	GPR55, Gα_q/11_, and Gα13 pathways, and their signaling via RhoA-ROCK as well as ERK1/2	Gangadharan et al., 2013
Hot plate test/rat	LPI	1 μg	Intra-PAG	Reduction in nociceptive threshold that is abolished by a pretreatment with ML-193, a GPR55 antagonist.	Pro-nociception mediated by GPR55 activation at central levels. Blockade of GPR55 signaling in the PAG may promote analgesia	Deliu et al., 2015


*CFA, Complete Freund’s Adjuvant; PNL, partial ligation of the sciatic nerve; CCI, chronic constriction injury; NAGly, *N*-arachidonylglycine; LPI, lysophosphatidylinositol; AA-5-HT, *N*-arachidonoyl-serotonin; WT, wild type; ACC, anterior cingulated cortex; PAG, periaqueductal gray; N/T, not tested.*

Nerve injury enhances expression of GPR18 mRNA in spinal cord and/or the DRG of rats, suggesting a potential role of GPR18 in the modulation of neuropathic pain (Malek et al., 2016). Accordingly, intrathecal administration of NAGly reduces mechanical allodynia in rats subjected to spinal nerve ligation and this effect is not prevented by pretreatment with either the CB_1_ or CB_2_ receptor antagonists AM251 and SR144528, respectively (Vuong et al., 2008). Although NAGly has been proposed as an endogenous GPR18 ligand, recent studies have found that resolvin D2 (RvD2) also activates GPR18 receptors (Chiang et al., 2015; Zhang et al., 2016). RvD2 activates recombinant human GPR18 in a receptor- and ligand-dependent manner and promotes the resolution of bacterial infections and organ protection (Chiang et al., 2015). Moreover, RvD2 enhances endothelial cell migration in a Rac-dependent manner *via* GPR18, and GPR18-deficient mice have an endogenous defect in perfusion recovery following hind limb ischemia (Zhang et al., 2016). In rodents, intrathecal administration of RvD2 reverses CFA-induced inflammatory pain, prevents formalin-induced spontaneous pain, and also reverses C-fiber stimulation-evoked long-term potentiation in the spinal cord (Park et al., 2011). However, RvD2 antinociceptive effects seem to be mediated by additional mechanisms involving the inhibition of transient receptor potential (TRPV1 and TRPA1) channels (Park et al., 2011). Undoubtedly, more studies to redefine the signaling pathways, ligands and physiological functions of GPR18 are needed.

GPR55 has been found highly expressed in large-diameter neurons, but present at low levels in small-diameter neurons of the mouse DRG (Lauckner et al., 2008). Indeed, reports suggest that GPR55 plays a role in modulating nociceptor excitability. Activation of GPR55 with lysophosphatidylinositol (LPI) promotes excitability in cultured large DRG neurons by increasing intracellular Ca^2+^ (Lauckner et al., 2008) and also produces mechanical hypersensitivity in mice after local peripheral administration (Gangadharan et al., 2013). Although there is a general consensus that LPI acts as an agonist for GPR55, it has been also reported that LPI modulates large-conductance Ca^2+^-activated potassium (K^+^) channels (BK_Ca_) (Bondarenko et al., 2011a,b), 2-pore domain potassium (K^+^)-channels (TREK-1) (Maingret et al., 2000; Danthi et al., 2003) and the potassium (K^+^) channel subfamily K member 4 (KCNK4 or TRAAK) (Maingret et al., 2000), transient receptor potential (TRPV2; Monet et al., 2009; Harada et al., 2017), and transient receptor potential (TRPM8; Vanden Abeele et al., 2006; Andersson et al., 2007) channels. All these channels are expressed in the primary nociceptive pathway and their activation either modulates or amplifies sensory information (Basbaum et al., 2009). Therefore, the pharmacological data with LPI should be taken with caution. Furthermore, LPI is not the sole GPR55 activator. The hydrophilic glycerophospholipid lyso-phosphatidyl-β-D-glucoside (LysoPtdGlc) was recently reported as a regulator of the nociceptive central axon projections by activating GPR55 with high affinity (Guy et al., 2015). This indicates that glycerophospholipids could play a role modulating nociceptive inputs *in vivo*.

Nerve damage increases GPR55 mRNA expression in the spinal cord and DRG of rats (Malek et al., 2016) suggesting the participation of these receptors in neuropathic pain. It has been shown that the synthetic GPR55 agonist O-1602 reduces movement-evoked firing of nociceptive C fibers in a rat model of acute joint inflammation, and this effect is blocked by the GPR55 receptor antagonist O-1918 (Schuelert and McDougall, 2011). O-1602 also has protective effects in a murine model of experimentally induced colitis, but this anti-inflammatory effect could not be mediated by GPR55 (Schicho et al., 2011).

On the other hand, other studies have reported that GPR55 knockout mice show a reduced tumor-induced mechanical hypersensitivity (Gangadharan et al., 2013). GPR55 agonist O-1602 produces pronociceptive effects in neuropathic rats (Breen et al., 2012). At the central nervous system, local injection of the GPR55 putative inverse agonist CID16020046 into the anterior cingulated cortex (ACC) produces antinociception in the formalin test by decreasing the extracellular signal-regulated kinase 1/2 (ERK1/2) phosphorylation in the ACC and c-*fos* mRNA expression in the spinal cord (Okine et al., 2016). Moreover, LPI administration into the periaqueductal gray (PAG) attenuates nociceptive latencies in a hot-plate test and also produces a concentration-dependent increase in intracellular Ca^2+^ levels in dissociated rat PAG neurons expressing GPR55 mRNA (Deliu et al., 2015). Although the exact mechanisms underlying the GPR55-mediated antinociceptive effects remain to be elucidated, it has been suggested that some cytokines (e.g., IL-4 and IL-10) are responsible for the modulatory effects observed during inflammatory pain conditions (Staton et al., 2008).

Using cell lines, other studies have shown that GPR55 couples to G*_α13_* and activates GTPases RhoA, Cdc42 and Rac1 (Ryberg et al., 2007; Henstridge et al., 2009). Some efforts have tried to elucidate the G-protein signaling pathway activated by GPR55 agonists *in vivo*. Using pharmacological and conditional genetic tools in mice, the research group headed by Rohini Kuner showed that LPI-mediated hypersensitivity depends on the activation of G*_α13_* and G*_αq/11_*, which in turn activate ERK1/2 (Gangadharan et al., 2013). In support of these results, it has been shown that LPI produces β-arrestin trafficking, MAPK, ERK1/2 phosphorylation and activates the G-protein signaling by a PKCβII-independent mechanism (Oka et al., 2007; Kapur et al., 2009). Interestingly, the effects on β-arrestin GPR55 complex formation, ERK1/2 phosphorylation and internalization of GPR55 are blocked by the GPR55 antagonist/partial agonist CP55,940 (Kapur et al., 2009), suggesting that a complex mechanism triggered upon GPR55 activation modulates G-coupled signaling pathways. Moreover, it has been documented that activation of GPR55 leads to additional p38 MAPK (Oka et al., 2010) and AKT phosphorylation (Pineiro et al., 2011). These events are related to the subsequent activation of several major transcription factors such as the nuclear factor of activated T-cells (NFAT) (Waldeck-Weiermair et al., 2008; Henstridge et al., 2009, 2010), CREB (Henstridge et al., 2010), NF-kB (Waldeck-Weiermair et al., 2008; Henstridge et al., 2010), and ATF2 (Oka et al., 2010).

Certainly, there is extensive literature indicating that signaling pathways involving MAPK and transcription factors such as NF-κB play an important role in pain (Niederberger and Geisslinger, 2008; Ji et al., 2009). However, it is worth emphasizing that most of the signaling mechanisms reported for GPR55 receptors have been obtained *in vitro* using cell lines and may not be completely translated to *in vivo* models. This is particularly important due to the recent discrepancies in the pain field using GPR55 knock-out mice. It was originally reported that mice lacking GPR55 show no differences in baseline pain responses compared to wild-type mice, but mechanical hyperalgesia is absent following either intraplantar CFA injection or partial nerve ligation (Staton et al., 2008). However, a recent study using knock-out mice suggests that GPR55 is dispensable for the development of inflammatory and neuropathic pain (Carey et al., 2017). According to these authors, GPR55 knock-out mice have no differences in mechanical, cold or heat hypersensitivity after intraplantar capsaicin, formalin or CFA injection. Likewise, development and maintenance of neuropathic pain after paclitaxel administration or partial nerve ligation is undistinguishable between GPR55 knock-out and wild-type mice. While the explanation for this discrepancy is not clear, Carey et al. have suggested that these differences could be due to multiple factors, including the way the GPR55 knock-out mice were made, the battery of tests used, freely moving animals versus restrained animals during the test, sex differences, body weight, and age of animals. Evidently, more behavioral studies using controlled experimental conditions will be necessary to define the importance of GPR55 receptors in modulating pain responses.

## Conclusion

Cannabinoids, *via* CB_1_ receptors, mainly induce inhibition of pain integration that seems to be useful particularly in the treatment of chronic pain, whereas CB_2_ stimulation mainly causes antiinflammation *via* negative modulation of the immune system. GPR18 and GPR55 have a role in integrating, transmitting and/or alleviating pain. However, further studies using more selective pharmacological tools combined with genetic tools to generate cell-specific ablation or reactivation of GPR18/GPR55 receptors in specific cell populations will help to clarify the functional role of these receptors to take advantage of them in therapeutics.

## Author Contributions

RG-A, PB-I, and EV-M developed the manuscript and discussed central ideas of it. AG-H and MC-L adapted the manuscript, designed graphs, and discussed central ideas of it. VG-S corrected the style and reviewed and edited the manuscript. MR supervised the project, worked on the conceptualization and acquired funding. BM-C conceived of the presented idea, integrated and edited information, and developed some central themes.

## Conflict of Interest Statement

The authors declare that the research was conducted in the absence of any commercial or financial relationships that could be construed as a potential conflict of interest.

## Footnotes

1 FAAH, Fatty Acid Amide Hydrolase Enzyme. FAAH is an integral membrane enzyme that hydrolyzes the endocannabinoid anandamide and related amidated signaling lipids. FAAH KO mice display elevated anandamide levels, showing reduced nociceptive transmission in several pain models. Journal of Neurobiology 61: 149–60.

2 http://www.utdallas.edu/bbs/painneurosciencelab/DRGtranscriptome/search.php
